# How does social presence influence public crisis information sharing intention? Situational pressure perspective

**DOI:** 10.3389/fpubh.2023.1124876

**Published:** 2023-07-11

**Authors:** Xiaoan Guo, Hengjiang Jin, Tianzhe Qi

**Affiliations:** ^1^School of Journalism, Chongqing University, Chongqing, China; ^2^Department of Journalism, School of Journalism and Communication, Northwest Minzu University for Nationalities, Lanzhou, Gansu, China

**Keywords:** social presence, risk perception, opinion climate, information sharing, situational pressure

## Abstract

**Objective:**

Public crises seriously affect social stability and personal health. When individuals are in a public crisis environment, they will have the impulse and intention to share information, which is a behavioral attitude shown in the face of a crisis. Public crisis information sharing intention will be affected by many factors. This study aims to examine how the process of social presence may influence information sharing intentions during a public crisis and the mediating effects of situational pressure, including risk perception of disease infection and consistency of perception of opinion climate.

**Methods:**

This was a cross-sectional study with 505 youth SNS users. In order to collect as suitable samples as possible, a research website was commissioned to conduct a questionnaire in the early stages of the COVID-19 pandemic in China. This questionnaire was utilized to measure social presence, risk perception of disease infection, consistency of perception of opinion climate and intention to share information about COVID-19. Structural equation modeling was used to examine variable relationships in the research model.

**Results:**

The results showed that social presence was significantly and positively associated with risk perception of disease infection (*B* = 0.42, *p* < 0.001), consistency of perception of opinion climate (*B* = 0.43, *p* < 0.001) and intention to share information about COVID-19 (*B* = 0.48, *p* < 0.001). Risk perception of disease infection (*B* = 0.19, *p* < 0.001) and consistency of perception of opinion climate (*B* = 0.18, *p* = 0.002) positively predicted youth SNS users’ intention to share information about COVID-19. Risk perception of disease infection and consistency of perception of opinion climate mediated the relationship between social presence and intention to share information about COVID-19 (*Z* = 2.66, CI: 0.03, 0.15; *Z* = 2.66, CI: 0.02, 0.16).

**Conclusion:**

The study further deepens our understanding of the mechanisms underlying social presence and information sharing intentions. These new findings suggest that some situational cues, including media environment factors (social presence) and perceived stress factors (risk perception of disease infection, consistency of perception of opinion climate) may influence information sharing intention. From the perspective of communication psychology, this study enriched the assessment of information sharing on social media and contributes to understanding of social presence and situation pressure, and it helps to provide specific references for effectively promoting netizens’ intention to share information about public crises.

## 1. Introduction

During the COVID-19 pandemic, people have become increasingly dependent on the internet for access to and the sharing of information (1). In particular, social media has become a dominant mode of communication (2), and COVID-19-related content is one of the major topics of discussion (3). Studies have shown that sharing COVID-19-related information on social networking services (SNS) can help individuals reach a consensus about the nature of the outbreak (4). Moreover, the sharing of truthful and accurate COVID-19 information can effectively address the negative social impacts of fake news (5) and help many people learn about COVID-19 during home quarantine. The sharing of information by individuals on social media and through interpersonal interaction can also affect the availability and accessibility of the information people need (6). In addition, scholars have suggested that a weak intention to share or no access to relevant information may hinder individual understanding of the pandemic (7). Research on COVID-19-related information sharing therefore remains essential.

Studies have suggested that information sharing is influenced by both personal and social factors. The former refers to personal attitudes, positions, and intentions, while the latter refers to social factors such as interpersonal relationships, social structures, social networks, and family structures (8, 9). Conceiving of information sharing as a subjective choice of individuals, these authors argue that such sharing can also be influenced by others and by society. However, several studies have explored issues related to COVID-19 such as disinformation and unverified information sharing (10–12) and emotions (13, 14). While these studies offer insights into the discussions and concerns around this global health crisis, few scholars have researched the information-sharing intention about COVID-19 itself.

In our view, the intention to share information about COVID-19 reflects individual choice considerations, which is more hidden than actual information-sharing behavior and thus requires further study and exploration. Moreover, while not as tangible as the information-sharing behavior, studying the intention to share information has practical implications. Not all people share information, and many remain in an underlying state of consciousness, which we believe is more critical because its hidden and uncertain nature is more challenging for the long-term stability of society and the response to COVID-19. Finally, research into the intention to share information can guide individuals in regard to awareness and psychology and propose measures to improve mental health and the perception of COVID-19. Consequently, it is important to study the intention to share information on COVID-19.

Currently, to prevent the adverse impact of COVID-19 on social stability and personal health, areas in China with a relatively higher case rate have adopted strict protective measures, asking residents to work and study from home and restricting travel and gatherings. As a result, people affected by the pandemic have turned to the internet for social and recreational activities that are not available offline. Tu (15) found that individuals use the media to perceive the presence of others and then develop a sense of social presence through online networking. Several authors have suggested that the concept of social presence is used to explain the impact of the media on communication behaviors (16). Information dissemination, as a behavioral manifestation of personal media use, permeates all online behaviors of individuals, enabling them to keep themselves informed about the COVID-19 pandemic while sharing relevant information. From this perspective, it is necessary to explore individuals’ perception of social presence in the process of information dissemination. In addition to the changes in the ways of interaction, the information environment of COVID-19 in China is under twofold situational pressure: stress, anxiety, and panic, mainly due to individuals’ perception of the risk of being infected (17); and the pressure of the public information environment in China, where individuals’ dissemination of information about the pandemic is influenced by public opinion, the media, friends, and strangers (18). Thus, it is useful to start from the concepts of social presence and situational pressure to explore the intention of the Chinese public to share information online during the COVID-19 pandemic.

## 2. Literature review

### 2.1. Stimulus-organism-response model

The S-O-R model, developed from the S-R (Stimulus–Response) theory, is a theoretical model of environmental influence on individual behavior proposed by Mehrabian and Russell (19), namely the “stimulus-body-response” model. The model assumes that the environment contains a variety of stimuli (S) that induce changes in people’s internal or organic state (O), which in turn lead to approach or avoidance behavior (R). The model posits that stimuli act on an individual’s decisions, intentions, or behaviors by influencing the organism’s (individual’s) cognition and emotions to promote its behavioral responses. The combination of these three conceptualizations constructs a formulaic theoretical model that includes indirect links between emotion and cognition, rather than a direct causal link between stimuli and actions (20). As one of the classic theories of cognitive psychology, the S-O-R model has been widely demonstrated in the fields of marketing, management, communication, and is used to study and explain user behavior. In the field of social media, some researchers focused on the perspective of media users, and discussed the impact of social media use on user emotions, behaviors or intentions through the S-O-R model (12, 21–23).

In the context of a global pandemic, we believe that the stimulus should be the state in which individuals perceive the “real” presence of others in the Internet environment. Zhu et al. (24) studying the relationship between online reviews and purchase intention, also mention social presence as a possible stimulus for online review generation. Because the widespread use of social media and the hyper-relational connectivity exhibited by social media make COVID-19 unique among previous epidemics, we focus on the presence effect of social media. To relate the organic aspects of S-O-R to the selected stimuli, we focus on social presence (25–27), which we believe leads to psychological and behavioral responses.

In addition, we use social information processing (SIP) theory to support the S-O-R framework, which emphasizes the influence of situational factors on people’s attitudes and behaviors (28). Situational cues, including stress perception (29) and peer comments (30) influence an individual’s online behavioral intentions. We believe that people’s risk perception of COVID-19 is a situational stressor of the disease in the early stage of the COVID-19 epidemic. In addition, when people are in the COVID-19 information environment, they are subject to public opinion environmental climate pressures from the media, government agencies, and other personal information. These two situational stressors may be important factors that influence people’s processing of COVID-19 information.

For the research model, using the S-O-R framework (19), we placed social presence as the environmental stimuli, two situational pressures (risk perception of disease infection and consistency of perception of opinion climate) as affecting the internal evaluation of the organism, and intention to share information as behavioral responses.

### 2.2. Intention to share information

The use of social media as a form of digital media consumption enables audiences to connect, communicate, and share information (31). Especially in the age of the mobile internet, the impacts of the widespread use of SNSs on people’s lives are obvious, of which the most significant is the nonlinear trend of the relationship between communicator and audience. In the age of social media, everyone can be both an audience and a communicator of information (32). Increasing numbers of people are sharing information or expressing their positions, attitudes, and views on social events or phenomena on social media (33).

The media acts as an important source of disease information, conveying a range of information that the public urgently needs, such as disease progression and treatment, policy interpretation, and personalized advice (34). Health information is closely related to people’s life and has become an important type of content that people seek for and share online. Several studies have pointed out that people are willing to share original or non-original health information with friends and strangers *via* social media (3, 12). The “privacy,” “openness,” “immediacy,” and “breadth” of social media greatly influence the intention and manner in which people share health information (35, 36).

### 2.3. Situational pressure

In previous research, scholars have pointed out that situational pressure is a key factor affecting individual behaviors. It is so named because most of this pressure comes from other people or situations, not from the individual (37). Here, we distinguish between two types of situational pressure: consistency in opinion climate perception and risk perception of disease infection.

#### 2.3.1. Risk perception of disease infection

Risk perception is at the heart of risk communication, and therefore the perceptibility of risk is both the core issue and the purpose of risk communication (38). As far as “risk” is concerned, it exists objectively while the perception of risk is subjective. Slovic (39) argues that risk perception refers to the public’s subjective judgment and perception of external objective risks. Authors such as Setbon, Raude, Fischler and Flahault (40) emphasize that situational responses to risk perception are subjective psychological behaviors to people or objects when individuals are at risk. Risk perception is also a commonly used concept in the field of health communication. Risk perception of COVID-19 infection, based on the individual’s risk judgment and perception, emphasizes the impact of environmental risk on an individual’s cognition and behavior (41–43).

Risk perception theory holds that people’s intentions and behaviors are not formed through simple, straightforward processes. In the decision-making process, people are influenced by various factors, especially the perception of potential risks (44, 45). For example, the Impersonal Impact Hypothesis (IMH) derived from risk perception theory, is a far-reaching theoretical hypothesis that has been widely applied to the field of journalism and communication. It argues that when individuals are at risk, they tend to compare the level of risk threat to others and themselves. The IMH theory also highlights the extent to which mass media and interpersonal communication channels have different impact on risk perception (46). The situation that created by news reports or media information has shown a greater impact on the generation and effectiveness of public perception of risk (37, 47).

Studies have shown that searching for information from different sources is a way to amplify personal risk perception and fear, which therefore contributes to information search (12, 48). When individuals are acutely aware of the danger they face, the pressure prevents them from assessing the validity of information and they rely on information from other people or different media sources to substantiate their judgment and feelings. The information provided in online media platforms plays an important moderating role in this process in order to help individuals to make an analytical opinion (49) and can have a positive impact on their response to public health crises (50). Additionally, it has been suggested that one of the key determinants of healthy behavior is an individual’s ability to perceive health risks (51). It can be inferred that a higher risk perception increases the public’s sensitivity, awareness, and need for risk information, augmenting the frequency of communication with others and the sharing of risk-related information with family and friends and prompting themselves or other people to take protective measures (52). We therefore posit the following hypothesis:

*Hypothesis 1*: The risk perception of disease infection is positively related to the intention to share information during the COVID-19 pandemic.

#### 2.3.2. Consistency of perception of opinion climate

The spiral of silence theory is the most representative one for explaining the perception of the opinion climate, which holds that individuals have the ability to perceive the climate of opinion (53, 54). People are more likely to express their personal opinions when their perceptions are those of the majority, and they choose to remain silent if they perceive the majority opinion to be different from theirs, out of fear of isolation (55). Thus, individuals under pressure continually assess the opinion climate (56, 57). Such continual assessment would enable one not only to keep abreast of the current opinion climate, but also to assess how it will develop in the future and then act accordingly (58).

The perception of the opinion climate refers to how an individual perceives the environment of public opinion. According to Salmon and Neuwirth (59), the perception of the opinion climate is not a single and homogenous concept, but may have different layers and should therefore not be measured simply or roughly. Rather, it should be measured according to the relationship between individuals and a “reference group,” which ranges from the media to the opinion climate within the groups that individuals interact with (59).

Consistency of perception of opinion climate refers to the degree to which people perceive their views as agreeing with those of others, such as opinions from the media, family, friends, and online strangers. Some studies have even suggested that the opinion pressure from the majority can even change the attitudes of the “few,” indicating that individual behavior is not exclusively rooted in one’s own mind (53, 60).

Earlier studies have suggested that the opinion climate can have a powerful effect on individual behaviors (61). Most scholars have a positive attitude toward whether the perception of the opinion climate can influence individuals’ behaviors (62, 63). Studies have also found that people with a greater fear of social isolation are more likely to keep up with specific sources of information about public opinion and be motivated to determine what others are thinking (18). During the COVID-19 pandemic, opinion climates have evolved on social media, consisting of the views of friends and family and online discussions with strangers. In a particular opinion climate, individuals may be fearful of social isolation or social attack for their inappropriate comments and are therefore more willing to share information consistent with the prevailing opinion. We now posit the following hypothesis:

*Hypothesis 2*: The consistency of perception of opinion climate is positively related to the intention to share information during the COVID-19 pandemic.

### 2.4. Social presence

As a key concept in social psychology, social presence refers to the extent to which an individual perceives the presence of others in the use of a medium that provides the individual with an experience close to a face-to-face interactive connection with others (64). An individual is perceived as a “real person” rather than a technological illusion. Biocca et al. (65) define social presence as a sense of “co-presence” between communicators and other individuals through media platforms, claiming that social presence can be a close relation between them from the perspective of relationship.

Studies have shown that the public has a strong sense of social presence in communities in SNSs (66), and one of the main motivations for joining virtual communities is information exchange (67). Thus, a sense of social presence may help to encourage forums and community members to immerse themselves in information sharing (68). In a study on information sharing among SNS users, researchers found that social presence may shape users’ intention to share information to a large extent (69). The perception of social presence of SNS users may help increase their intention to share information about the COVID-19 pandemic. We therefore posit the following hypothesis:

*Hypothesis 3*: Social presence is positively related to the intention to share information during the COVID-19 pandemic.

Risk perception, as a concept of social psychology, is the public’s cognitive and psychological response to a threatening situation and event (40). In the process, risk perception of the public is influenced by the “pseudo-environment” constructed by the media: media coverage and the information environment may create by others and institutions on media platforms contribute significantly to the public’s risk perception (37). Studies have shown that social media has become a main way for the public to access risk information (70) and that high exposure to risk-related information on media platforms can increase the level of risk perception (41, 47, 71). Thus, instead of facing risk directly, people may perceive risk based on the “virtual environment” created by the media (39). The media may create an environment of public opinion that has a social presence effect. People’s involvement in risky events is positively correlated with the perceived risk associated with the event (72, 73), and the stronger the sense of social presence, the more individuals tend to perceive the risk as being closer to them, thus inducing stronger behavioral intentions for risk prevention (74). We therefore posit the following hypothesis:

*Hypothesis 4*: Social presence is positively related to the risk perception of disease infection during the COVID-19 pandemic.

**Figure 1:**
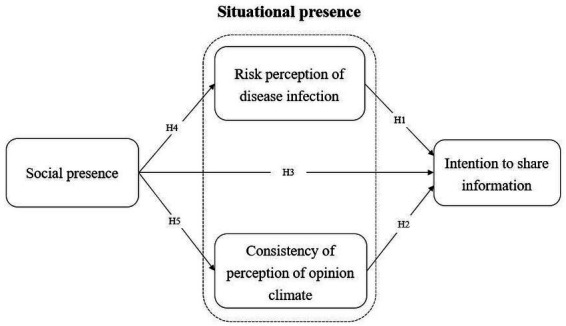
Hypothesized model of the research framework.

Biocca, Harms, and Burgoon (75) note that it is useful to assess the similarities and differences of social presence between media, as different media situations leading to diverse social presence perceptions. These perceptions are influenced by media characteristics such as interactivity, the abundance of nonverbal information, suggesting that the social presence theory can reflect the communication effect of varied media (65, 74). Obviously, there is a dependency between social presence and the communication situation. As for specific media, studies have shown that the loss of nonverbal information and anonymity (such as facial expressions) in computer-mediated communication (CMC) environments may reduce the perception of social presence, and thus can relieve the tension of the public in networking as well as the pressure brought by unanimous opinions (76). Here, it is apparent that the degree of social presence affects a group’s perception of social pressure and normative. In China, SNS platforms (such as We-chat, Weibo and short-video platforms) allow users to communicate anonymously and express themselves through voice messages, video calls or memes. In this communication environment of high context, the users’ perception of social presence and their ability to perceive the consistency in opinion climate are enhanced (Figure 1). We therefore make the following hypothesis:

*Hypothesis 5*: Social presence is positively related to the consistency of perception of opinion climate during the COVID-19 pandemic.

## 3. Research methodology

### 3.1. Data collection

Since the target population of this study are social networking site users, in order to collect as suitable a sample as possible, a research website was commissioned to conduct a questionnaire in the early stages of the COVID-19 pandemic in China. After the questionnaire is entered into the platform, the platform selects the SNS users in the sample database, and pushes the questionnaire to the target subjects through simple random sampling. Participants were recruited from an online questionnaire platform^1^ between July 15 and July 30, 2020, for a 15-min online questionnaire with a compensation of $1.40. In total, 897 individuals took the questionnaire, and 505 were retained in the final sample. The excluded participants (1) failed to select the designated answer for any of three attention check questions throughout the questionnaire, or (2) spent less than 5 min with the questionnaire, which should take at least 10 min based on our pilot test. (3) Due to the small number of respondents aged 45 and above (*N* = 11), the data of respondents aged 45 and above were removed. Therefore, participants in this study were 14–44 year olds who used SNS. According to the 50^th^ Statistical Report on China’s Internet Development, there are approximately 737 million netizens aged from 10 to 49 years old in China, accounting for 70.1% of total netizens (77). This data suggest that most Chinese youths are netizens. Table 1 presents the demographic information of the respondents.

**Table 1:** Demographic information of the respondents (*N* = 505).

Demographics	*n* (%)
**Gender**	
Male	210 (41.6)
Female	295 (58.4)
**Age groups (years)**	
14–19	69 (13.7)
20–29	219 (43.4)
30–39	135 (26.7)
40–44	82 (16.2)
Age (years), mean (SD)	27.9 (8.4)
**Education**	
High school or below	84 (16.6)
Two-year College	178 (35.2)
Four-year College	138 (27.3)
Graduate school or above	105 (20.8)
**Employment status**	
Employed full time	307 (60.8)
Student	106 (20.9)
Self-employed	64 (12.7)
Unemployed or retired	19 (3.8)
Others	9 (1.8)

### 3.2. Measurement

To ensure the validity of the constructs, the measurement of all variables was adjusted from the established studies to the background of the present study (see Table 2). All items were measured on Likert scales (1 = *strongly disagree*, 5 = *strongly agree*). The six items for measuring *social presence* were identified based on the research by Lu, Fan, and Zhou (25) and Gao et al. (66). *Risk perception of disease infection* was measured with three items according to the studies by Lin and Lagoe (78). *Consistency of perception of opinion climate* was measured with three items based on the studies of Salwen et al. (79). The measure of *intention to share information* consists of six items and was built upon the work of Zhang et al. (80) and Shang et al. (81).

**Table 2:** Measurement items.

Item	Loading
**Social presence**	
SP1. On SNS, I can perceive the presence of others	0.72
SP2. SNS gives me an immersive feeling	0.72
SP3. SNS is like a person to me, it feels real	0.85
SP4. SNS makes me feel warm	0.86
SP5. On SNS, I feel a kinship	0.72
SP6. The intimacy can be felt on SNS	0.76
**Risk perception of disease infection**	
RP1. If an SNS user is diagnosed with COVID-19, I will perceive a greater risk of the pandemic	0.77
RP2. The spread of COVID-19 makes it seem likely that anyone can get an infection	0.78
RP3. I will suspect myself of having COVID-19 if I have symptoms similar to those diagnosed with COVID-19	0.62
**Consistency of perception of opinion climate**	
OC1. My opinions on the COVID-19 pandemic echoes what that of mainstream media	0.70
OC2. My friends and I share same views on the COVID-19 pandemic	0.62
OC3. My thoughts on the COVID-19 pandemic are with most users	0.81
**Intention to share information**	
ISI1. I would like to discuss the COVID-19 pandemic on SNS	0.77
ISI2. On SNS, I would like to share information about the COVID-19 pandemic	0.77
ISI3. I would like to initiate/participate in activities of knowledge sharing about COVID-19 on SNS	0.71
ISI4. I would like to share my thoughts on the COVID-19 pandemic on SNS	0.76
ISI5. I would to repost information from other SNS users about the COVID-19 pandemic	0.81
ISI6. I am willing to forward information of the COVID-19 pandemic to SNS groups	0.71

## 4. Data analysis and results

### 4.1. Measurement model

Following conventional guidelines (82), a series of confirmatory factor analyses (CFA) was conducted to obtain a proper measurement model. The final CFA model fitted the data with *χ*^2^(129) = 305.73, GFI = 0.935, AGFI = 0.914, RMSEA = 0.052, TLI = 0.951, CFI = 0.935, IFI = 0.959. The measurement model was evaluated using indicators of reliability and validity. Reliability of the constructs was tested with Cronbach’s α and composition reliability (CR) values, which should be above 0.700 (83). Cronbach’s α values ranged from 0.75 to 0.90 and CR values from 0.76 to 0.90, indicating a good reliability in all constructs, as shown in Table 3.

**Table 3:** Descriptive statistics and reliability.

	Mean	SD	Cronbach’s α	CR	AVE
Social presence	3.23	0.78	0.90	0.90	0.60
Risk perception of disease infection	3.08	0.87	0.77	0.77	0.53
Consistency of perception of opinion climate	3.66	0.72	0.75	0.76	0.51
Intention to share information	3.00	0.90	0.89	0.89	0.57

Convergent validity was evaluated by average variance extracted (AVE). Table 3 shows that the AVE values of the constructs range between 0.51 and 0.60, which are above the standard of 0.50 (83), indicating a good convergent validity. The discretionary validity was assessed through the relation between the square root of the AVE of each construct and the value of the corresponding correlation matrix square (83). Table 4 shows that the square root of the AVE of each construct is greater than the value of the corresponding correlation matrix square, suggesting adequate discriminant validity.

**Table 4:** Construct correlations.

	1	2	3	4
1. Social presence	**0.77**			
2. Risk perception of disease infection	0.36	**0.73**		
3. Consistency of perception of opinion climate	0.39	0.42	**0.72**	
4. Intention to share information	0.53	0.38	0.39	**0.75**

The diagonal elements represent the square root of AVE.

### 4.2. Structural model

As Table 5 shows, the resulting model fit indices suggest an acceptable fit (84, 85). Most indices are above their ideal criterion levels: *χ*^2^(130) = 336.65, GFI = 0.929, AGFI = 0.907, RMSEA = 0.056, TLI = 0.944, CFI = 0.952, IFI = 0.952.

**Table 5:** Model fit of research model.

Model fit index	Criterion	Model fit of research model	Fit
MLc2	Smaller is better	336.65	Ideal
DF (degree of freedom)	Bigger is better	130	Ideal
Normed Chi-square (c2/DF)	1 < c2/DF < 3	2.59	Ideal
GFI	>0.9	0.929	Ideal
AGFI	>0.9	0.907	Ideal
RMSEA	<0.08	0.056	Ideal
TLI(NNFI)	>0.9	0.944	Ideal
CFI	>0.9	0.952	Ideal
IFI	>0.9	0.952	Ideal

The structural model was evaluated using the coefficient of determination (*R*^2^) and the significance of each path coefficient. The data analysis showed that social presence accounted for 13.6% of the variations in risk perception of disease infection and 16.6% of the variations in consistency of perception of opinion climate. Social presence, risk perception of disease infection, and consistency of perception of opinion climate explained 33.6% of the variation in the intention to share information.

**Figure 2:**
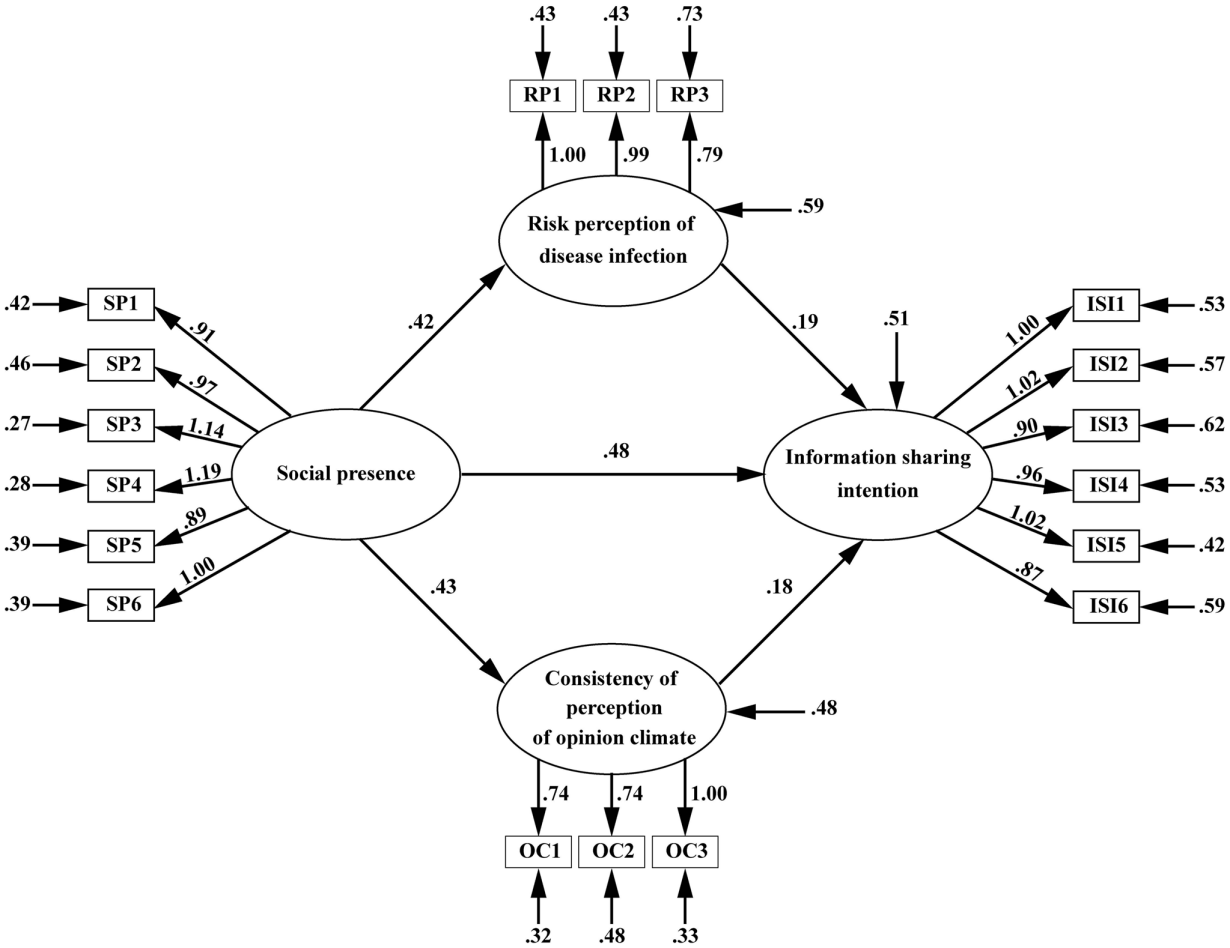
Path analysis and results.

In terms of path coefficients, social presence positively predicted risk perception of disease infection (*B* = 0.42, *p* < 0.001) and consistency of perception of opinion climate (*B* = 0.43, *p* < 0.001). SNS users with higher perception of social presence seemed to have a higher risk perception of COVID-19 infection and a greater consistency of perception of opinion climate. Thus, *H4* and *H5*was supported. Social presence has a significant impact on SNS users’ intention to share information about COVID-19 (*B* = 0.49, *p* < 0.001), suggesting that when social presence increased, SNS users were more willing to share information about COVID-19. Thus, *H3* was supported. Risk perception of disease infection (*B* = 0.19, *p* < 0.001) and consistency of perception of opinion climate (*B* = 0.18, *p* = 0.002) positively predicted SNS users’ intention to share information about COVID-19, implying that the higher the risk perception or the consistency of perception of opinion climate, the greater their intention to share information about COVID-19. Thus, *H1* and *H2* was supported (Figure 2 and Table 6).

**Table 6:** Hypothesis testing results.

Hypotheses	Path coefficient	*t* value	*p* value	Conclusion
RP--- > ISI (*H1*)	0.19	3.53	<0.001	Supported
OC--- > ISI (*H2*)	0.18	3.04	0.002	Supported
SP--- > ISI (*H3*)	0.48	7.18	<0.001	Supported
SP--- > RP (*H4*)	0.42	6.82	<0.001	Supported
SP--- > OC (*H5*)	0.43	7.53	<0.001	Supported

Path coefficient is unstandardized estimates.

### 4.3. Mediation analysis

To further analyze how social presence influences the intention to share information, we examined the mediation effects of risk perception of disease infection and consistency of perception of opinion climate. We performed an analysis on mediation effects using the bootstrap method, which involves 5,000 iterations with a 95% confidence interval (86).

As Table 7 shows, under the 95% confidence interval, the total effect is 4, the direct effect is 6.67, 6.5, 6.83, 3, 2.66 and the mediation effect is 2.66, 2.66, respectively, and the Z value is greater than 1.96, indicating that the mediation model is significant. of the eight estimated intervals shown by the bootstrap, none of the interval values contained 0, indicating a significant established confidence interval of 95%.

**Table 7:** Result of mediation effects test (bootstrap 5,000 samples).

Path	Point estimate	Product of coefficients		Bootstrapping	
		SE	Z	Bias-Corrected 95% CI	Percentile 95% CI
				**Total effects**	
SP → ISI	0.16	0.04	4	[0.09, 0.26]	[0.08, 0.25]
				**Direct effects**	
SP → ISI	0.40	0.06	6.67	[0.28, 0.52]	[0.28, 0.52]
SP → RP	0.39	0.06	6.50	[0.25, 0.48]	[0.25, 0.48]
SP → OC	0.41	0.06	6.83	[0.28, 0.52]	[0.28, 0.52]
RP → ISI	0.18	0.06	3	[0.06, 0.30]	[0.06, 0.30]
OC → ISI	0.16	0.06	2.66	[0.04, 0.29]	[0.03, 0.28]
				**Mediation effects**	
SP → RP → ISI	0.08	0.03	2.66	[0.03, 0.15]	[0.03, 0.15]
SP → OC → ISI	0.08	0.03	2.66	[0.02, 0.16]	[0.02, 0.14]

When social presence is an independent variable, “risk perception of disease infection” and “consistency of perception of opinion climate” have mediating effects on “the intention to share COVID-19 information.”

## 5. Discussion and implications

### 5.1. Discussion

The results confirmed that social presence is a positive predictor of risk perception of disease infection, consistency of perception of opinion climate, and intention to share information of SNS users in China, further suggesting that when using new media technologies, the perception of social presence puts SNS users under situational pressure and influences their behaviors. The functions of SNS as an information sharing tool have been of continuing interest to researchers (87). It has been pointed out that there are several underlying reasons users share information online (69). COVID-19 as a “booster” for information sharing (88, 89) has sparked a global passion for discussing the pandemic.

During the COVID-19 pandemic, social media platforms have been a key channel for the Chinese public to stay connected (90). The contact with others achieved through a certain medium has given the Chinese public a strong sense of social presence. Hassanein et al. (91) note the expansion of social presence, which, as a hallmark of the medium, can be used to measure media sensitivity, intimacy, and friendliness. For SNS users, a sense of intimacy and co-presence on the platform can lead to a psychological involvement and participation that, while virtual, feels like being in a real environment, improving their situational perception. Moreover, Lin et al. (69) expects that users with a higher sense of social presence are more willing to post and share information and knowledge, more likely to receive social support, and to respond to others’ information requests, leading to more positive attitudes toward information sharing. Our research findings support this view. We argue that after a public crisis, everyone is in a panic mood and expects to learn about the social impact of the crisis and coping strategies from social media, so the demand and sharing expectations for crisis information are higher. Therefore, in the context of social media, social presence not only makes social media an effective place for users to connect with each other (92, 93), but social presence can also be an important media attribute that affects high or low information sharing intentions of users.

Finally, we found that the risk perception of disease infection and the consistency of perception of opinion climate can positively predict the intention of Chinese SNS users to share information, implying that their sharing of information about COVID-19 did not happen randomly. Previous studies have pointed to the complexity of people’s decision-making (94). Our study shows that Chinese SNS users are affected by situational pressure when sharing information about COVID-19 on social media, which is also driven by psychosocial factors. As people are more likely to be alerted to risks of the pandemic because of health concerns, this risk perception drives SNS users’ intent to share more of the information they receive to help more people avoid risk.

Furthermore, during COVID-19, news reports in domestic and foreign media, opinions of intellectuals or professional institutions, information from and opinions of acquaintances and strangers have jointly created an opinion climate on Chinese social media platforms. There are two main views on the impact of the online opinion climate on individuals’ expression of opinion: one claims a weak influence, suggesting that perception of the opinion climate has little effect on the expression of opinion (54, 95, 96), while the other claims that the perception of the opinion climate can greatly affect the expression of individual opinions (58, 60). Our findings have echoed the latter views. In a public opinion climate in which the Chinese government tightly controls media coverage and social media, a consistent online opinion environment has been created. Perceiving the pressure of a mainstream opinion climate, SNS users tend to express views that align with the mainstream.

While conclusions are drawn above, they still work under the actual situations that individuals faced in the Chinese context. When individuals are outside a crisis situation, they may choose to express views that are in line with the prevailing view out of self-preservation and security concerns, under the pressure of consistency with the opinion climate. However, when individuals are threatened by COVID-19, their immediate interests and relationships are compromised.

In this circumstance, although people are aware of the prevailing public opinion climate, they also tend to break the consistency and express personal experiences and feelings in the hope that their claims will receive public attention and support, thus forming an “inverse spiral of silence” (97) in China’s online environment, influencing the opinion climate, generating non-mainstream information, and gradually changing mainstream views. As well as testing the mechanism of the “spiral of silence” in which individuals tend to exhibit self-favorable behaviors when relational connections are weak, we also suggest that when the high perception of social presence on SNS is related to personal experience and interests, their information that is inconsistent with the prevailing opinion climate will resonate strongly with the public, who may support the spread of non-mainstream views, prompting policy changes and benign social development.

### 5.2. Theoretical and managerial implications

This study has produced some theoretical implications. Some studies have explored COVID19 information sharing based on use and satisfaction theory determined by different motivations, such as altruism (98), social interactivity (99), and instant news sharing (10). These motivational factors are a reflection of the individual’s sense of subjectivity and needs. Our study, however, distinguishes itself from this tendency by discussing COVID19 information sharing intentions in terms of the psychology of new media use and individual perceived contextual factors, focusing on information sharing from the perspective of media environment perception, and further explains the mechanism of social presence on information sharing intention, discusses the mediating role of situational pressure, and constructs a model of media environment perception for information sharing intention research.

This study has some important implications. We found that social presence is a positive influence on the intention to share information about a public crisis. We argue that social media has a different presence effect, with social media acting as a means of social mobilization and a “stress valve” to reduce pandemic-induced tensions and fears (100). Therefore, coping with public social emotions and behavioral intentions brought about by public crises can be considered effective by using the social media presence effect. Secondly, the findings show that there is a positive relationship between social presence and risk perception of disease infection and consistency of perception of opinion climate, which suggests that in public crisis events, in addition to the impact of information (54, 101–104), we should also pay attention to the psychological perception of SNS users’ media use factors that affect people’s risk perception and opinion climate perception. Finally, our study also found that risk perception of disease infection and consistency of perception of opinion climate can effectively influence information sharing intention. This reflects the need for public crisis management processes to pay attention to the quality of information and the opinion pressure generated by the information.

### 5.3. Limitations and direction for future research

Several limitations of the study should be acknowledged. The study failed to distinguish or track people’s intention to share information at different stages of the development of COVID-19. After nearly 3 years of COVID-19, changes must have taken place in public thinking and behavior as a result of the response to the pandemic. Subsequent studies could use text-tracking to explore differences in the intention to share information and the differences in its impacts on users at different stages of COVID-19. Our fundamental concern is the intention to share information, but the discussion of information-sharing or non-sharing behaviors remains to be explored, and more detailed discussions about sharing disinformation or unverified information about COVID-19 (10–12) and different types of information sharing (e.g., circle of acquaintances, the community of strangers) may be fruitful for future research. In addition, information sharing intention is the psychological willingness of personal behavior, which may not necessarily transfer to indeed sharing information. Finally, our research samples were collected from the research website. Therefore, the sampling population comes from the SNS users of the research website sample database, and the obtained data is not a national representative sample. However, the conclusions obtained by the research focusing on SNS users can still show that social presence has a positive predictive effect on the intention to share information about public crises, and the research is instructive. On this basis, future research can expand the scope of the overall sample or adjust the sample proportion to collect a nationally representative sample. In addition, it is also possible to focus on a certain group (such as youth or the older adult) to discover differences between samples.

## 6. Conclusion

Risk perception of disease infection and consistency of perception of opinion climate mediated the relationship between social presence and intention to share information about COVID-19. Findings suggest that some situational cues, including media environment factors (social presence) and perceived stress factors (risk perception of disease infection, consistency of perception of opinion climate) may influence information sharing intention. From a communication psychology perspective, this study enriches the assessment of social media information sharing, contributes to the understanding of social presence and situational pressure, and helps to provide specific references for effectively promoting netizens’ intention to share information about public crises.

## Data availability statement

The original contributions presented in the study are included in the article/Supplementary material, further inquiries can be directed to the corresponding author.

## Ethics statement

The studies involving human participants were reviewed and approved by Institutional Review Board Statement: The study was conducted according to the guidelines of the Declaration of Helsinki, and approved by the Academic Board of the School of Journalism and communication, Chongqing University (4 May 2020 of approval). Informed Consent Statement: Informed consent was obtained from all subjects involved in the study. Written informed consent to participate in this study was provided by the participants’ legal guardian/next of kin.

## Author contributions

XG and HJ: conceptualization. HJ: data curation, formal analysis, methodology, and writing – original draft. XG: funding acquisition and project administration. HJ and TQ: investigation. XG, HJ, and TQ: writing – review and editing. All authors contributed to the article and approved the submitted version.

## Funding

This survey was approved by the National Social Science Fund of China (no. 22BXW15) and Fundamental Research Fund for the Central Universities (no. 2020CDJSK07PY23).

## Conflict of interest

The authors declare that the research was conducted in the absence of any commercial or financial relationships that could be construed as a potential conflict of interest.

## Publisher’s note

All claims expressed in this article are solely those of the authors and do not necessarily represent those of their affiliated organizations, or those of the publisher, the editors and the reviewers. Any product that may be evaluated in this article, or claim that may be made by its manufacturer, is not guaranteed or endorsed by the publisher.

## Footnotes

^1^ www.wjx.cn

## References

[1] Van Aelst P Toth F Castro L Štětka V Vreese CD Aalberg T. Does a crisis change news habits? A comparative study of the effects of COVID-19 on news media use in 17 European countries. Digit Journal. (2021) 9:1208–38. doi: 10.1080/21670811.2021.1943481

[2] London J Matthews K. Crisis communication on social media - lessons from Covid-19. J Decis Syst. (2021) 31:150–70. doi: 10.1080/12460125.2021.1926612

[3] Kirkpatrick AW Park M Domgaard S Zhao W Steinberg C Hsu YC. Vaccine videos and information sharing: the effects of framing, evidence type, and speaker expertise. J Health Commun. (2021) 26:608–17. doi: 10.1080/10810730.2021.1983892 34596481

[4] Zheng H Goh DHL Lee EWJ Lee CS Theng YL. Understanding the effects of message cues on COVID-19 information sharing on twitter. J Assoc Inf Sci Technol. (2022) 73:847–62. doi: 10.1002/asi.24587 34901313 PMC8653370

[5] Laato S Islam AKMN Islam MN Whelan E. What drives unverified information sharing and cyberchondria during the COVID-19 pandemic? Eur J Inf Syst. (2020) 29:288–305. doi: 10.1080/0960085X.2020.1770632

[6] Lu L Liu J Yuan YC Burns KS Li D. Source trust and covid-19 information sharing: the mediating roles of emotions and beliefs about sharing. Health Educ Behav. (2020) 48:132–9. doi: 10.1177/1090198120984760 33356578 PMC8685569

[7] Nelson S Abimbola S Jenkins A Naivalu K Negin J. Information sharing, collaboration, and decision-making during disease outbreaks: the experience of Fiji. J Decis Syst. (2021) 31:171–88. doi: 10.1080/12460125.2021.1927486

[8] Wang W Zhuang X Shao P. Exploring health information sharing behavior of chinese elderly adults on wechat. Healthcare. (2020) 8:e207. doi: 10.3390/HEALTHCARE8030207 PMC7550995 32664219

[9] Wu X Kuang W. Exploring influence factors of WeChat users’ health information sharing behavior: based on an integrated model of TPB, UGT and SCT. Int J Hum Comput Interact. (2021) 37:1243–55. doi: 10.1080/10447318.2021.1876358

[10] Apuke OD Omar B. Fake news and COVID-19: modelling the predictors of fake news sharing among social media users. Telematics Inform. (2021) 56:101475. doi: 10.1016/j.tele.2020.101475 PMC7390799 34887612

[11] Huang Q Lei S Ni B. Perceived information overload and unverified information sharing on WeChat amid the COVID-19 pandemic: a moderated mediation model of anxiety and perceived herd. Front Psychol. (2022) 13:837820. doi: 10.3389/fpsyg.2022.837820 35185742 PMC8853730

[12] Laato S Islam AN Farooq A Dhir A. Unusual purchasing behavior during the early stages of the COVID-19 pandemic: the stimulus-organism-response approach. J Retail Consum Serv. (2020) 57:102224. doi: 10.1016/j.jretconser.2020.102224

[13] Allen B. Emotion and covid-19: toward an equitable pandemic response. J Bioethical Inquiry. (2021) 18:403–6. doi: 10.1007/s11673-021-10120-4 34463911 PMC8406008

[14] Li MH Chen Z Rao LL. Emotion, analytic thinking and susceptibility to misinformation during the covid-19 outbreak. Comput Hum Behav. (2022) 133:107295. doi: 10.1016/j.chb.2022.107295 PMC8991995 35431427

[15] Tu C-H. The measurement of social presence in an online learning environment. Int J E Learn. (2002) 1:34–45. doi: 10.17471/2499-4324/421

[16] Shen KN Khalifa M. Exploring multidimensional conceptualization of social presence in the context of online communities. Int J Hum Comput Interaction. (2008) 24:722–48. doi: 10.1080/10447310802335789

[17] Ho MC Shaw D Lin S Chiu YC. How do disaster characteristics influence risk perception? Risk Anal. (2008) 28:635–43. doi: 10.1111/J.1539-6924.2008.01040.X 18643821

[18] Hayes AF Matthes J Eveland WP. Stimulating the quasistatistical organ: fear of social isolation motivates the quest for knowledge of the opinion climate. Commun Res. (2013) 40:439–62. doi: 10.1177/0093650211428608

[19] Mehrabian A Russell JA. An approach to Environmental Psychology. Cambridge, Mass: The MIT Press (1974).

[20] Xu J Benbasat I Cenfetelli RT. The nature and consequences of trade-off transparency in the context of recommendation agents. MIS Q. (2014) 38:379–406. doi: 10.25300/MISQ/2014/38.2.03

[21] Cao X Sun J. Exploring the effect of overload on the discontinuous intention of social media users: an SOR perspective. Comput Hum Behav. (2018) 81:10–8. doi: 10.1016/j.chb.2017.11.035

[22] Carlson J Rahman M Voola R De Vries N. Customer engagement behaviours in social media: capturing innovation opportunities. J Serv Mark. (2018) 32:83–94. doi: 10.1108/JSM-02-2017-0059

[23] Luqman A Cao X Ali A Masood A Yu L. Empirical investigation of Facebook discontinues usage intentions based on SOR paradigm. Comput Hum Behav. (2017) 70:544–55. doi: 10.1016/j.chb.2017.01.020

[24] Zhu L Li H Wang FK He W Tian Z. How online reviews affect purchase intention: a new model based on the stimulus-organism-response (SOR) framework. Aslib J Inf Manag. (2020) 72:463–88. doi: 10.1108/AJIM-11-2019-0308

[25] Lu B Fan W Zhou M. Social presence, trust, and social commerce purchase intention: an empirical research. Comput Hum Behav. (2016) 56:225–37. doi: 10.1016/J.CHB.2015.11.057

[26] Ming J Jianqiu Z Bilal M Akram U Fan M. How social presence influences impulse buying behavior in live streaming commerce? The role of SOR theory. Int J Web Inform Syst. (2021) 17:300–20. doi: 10.1108/IJWIS-02-2021-0012

[27] Yasir A Hu X Ahmad M Rauf A Shi J Ali Nasir S. Modeling impact of word of mouth and E-government on online social presence during COVID-19 outbreak: a multi-mediation approach. Int J Environ Res Public Health. (2020) 17:2954. doi: 10.3390/ijerph17082954 32344770 PMC7216275

[28] Salancik GR Pfeffer J. A social information processing approach to job attitudes and task design. Adm Sci Q. (1978) 23:224–53. doi: 10.2307/2392563 10307892

[29] Picazo-Vela S Chou SY Melcher AJ Pearson JM. Why provide an online review? An extended theory of planned behavior and the role of big-five personality traits. Comput Hum Behav. (2010) 26:685–96. doi: 10.1016/j.chb.2010.01.005

[30] Moe WW Schweidel DA. Online product opinions: incidence, evaluation, and evolution. Mark Sci. (2012) 31:372–86. doi: 10.1287/mksc.1110.0662

[31] Correa T Hinsley AW de Zúñiga HG. Who interacts on the web?: the intersection of users' personality and social media use. Comput Hum Behav. (2010) 26:247–53. doi: 10.1016/J.CHB.2009.09.003

[32] Wall M. Citizen journalism: a retrospective on what we know, an agenda for what we don’t. Digit Journal. (2015) 3:797–813. doi: 10.1080/21670811.2014.1002513

[33] Shibchurn J Yan X. Information disclosure on social networking sites: an intrinsic-extrinsic motivation perspective. Comput Hum Behav. (2015) 44:103–17. doi: 10.1016/J.CHB.2014.10.059

[34] Hilton S Hunt K. UK newspapers' representations of the 2009-10 outbreak of swine flu: one health scare not over-hyped by the media? J Epidemiol Community Health. (2011) 65:941–6. doi: 10.1136/JECH.2010.119875 21131303 PMC3171979

[35] AlQarni ZA Yunus F Househ MS. Health information sharing on Facebook: an exploratory study on diabetes mellitus. J Infect Public Health. (2016) 9:708–12. doi: 10.1016/J.JIPH.2016.08.015 27618634

[36] Lin HC Chen YJ Chen CC Ho WH. Expectations of social networking site users who share and acquire health-related information. Comput Electr Eng. (2018) 69:808–14. doi: 10.1016/J.COMPELECENG.2018.02.014

[37] He G Mol APJ Zhang L Lu Y. Nuclear power in China after Fukushima: understanding public knowledge, attitudes, and trust. J Risk Res. (2014) 17:435–51. doi: 10.1080/13669877.2012.726251

[38] Aakko E. Risk communication, risk perception, and public health. Wis Med J. (2004) 103:25–7. PMID: 15101463

[39] Slovic P. Perception of risk. Science. (1987) 236:280–5. doi: 10.1126/SCIENCE.3563507 3563507

[40] Setbon M Raude J Fischler C Flahault A. Risk perception of the "mad cow disease" in France: determinants and consequences. Risk Anal. (2005) 25:813–26. doi: 10.1111/J.1539-6924.2005.00634.X 16268931

[41] Abrams EM Greenhawt M. Risk communication during COVID-19. The journal of allergy and clinical immunology. In Pract. (2020) 8:1791–4. doi: 10.1016/j.jaip.2020.04.012 PMC7158804 32304834

[42] Oh SH Lee SY Han C. The effects of social media use on preventive behaviors during infectious disease outbreaks: the mediating role of self-relevant emotions and public risk perception. Health Commun. (2021) 36:972–81. doi: 10.1080/10410236.2020.1724639 32064932

[43] Schneider CR Dryhurst S Kerr J Freeman AL Recchia G Spiegelhalter D. COVID-19 risk perception: a longitudinal analysis of its predictors and associations with health protective behaviours in the United Kingdom. J Risk Res. (2021) 24:294–313. doi: 10.1080/13669877.2021.1890637

[44] Caserotti M Girardi P Rubaltelli E Tasso A Lotto L Gavaruzzi T. Associations of COVID-19 risk perception with vaccine hesitancy over time for Italian residents. Soc Sci Med. (2021) 272:113688. doi: 10.1016/j.socscimed.2021.113688 33485215 PMC7788320

[45] Lim N. Consumers' perceived risk: sources versus consequences. Electron Commer Res Appl. (2003) 2:216–28. doi: 10.1016/S1567-4223(03)00025-5

[46] Tyler TR Cook FL. The mass media and judgments of risk: distinguishing impact on personal and societal level judgments. J Pers Soc Psychol. (1984) 47:693–708. doi: 10.1037/0022-3514.47.4.693

[47] Malecki KM Keating JA Safdar N. Crisis communication and public perception of COVID-19 risk in the era of social media. Clin Infect Dis. (2021) 72:697–702. doi: 10.1093/cid/ciaa758 32544242 PMC7337650

[48] Soroya SH Farooq A Mahmood K Isoaho J Zara SE. From information seeking to information avoidance: understanding the health information behavior during a global health crisis. Inf Process Manag. (2021) 58:102440. doi: 10.1016/j.ipm.2020.102440 33281273 PMC7700063

[49] Case DO. Looking for Information: A Survey of Research Information Seeking, Needs and Behavior. 3rd ed. Bingley: Emerald Group Publishing (2012).

[50] Kittler AF Hobbs J Volk LA Kreps GL Bates DW. The internet as a vehicle to communicate health information during a public health emergency: a survey analysis involving the anthrax scare of 2001. J Med Internet Res. (2004) 6:99–110. doi: 10.2196/JMIR.6.1.E8 PMC1550585 15111274

[51] Ferrer RA Klein WMP. Risk perceptions and health behavior. Curr Opin Psychol. (2015) 5:85–9. doi: 10.1016/J.COPSYC.2015.03.012 26258160 PMC4525709

[52] Lindell MK Perry RW. The protective action decision model: theoretical modifications and additional evidence. Risk Anal. (2012) 32:616–32. doi: 10.1111/J.1539-6924.2011.01647.X 21689129

[53] Noelle-Neumann E. The spiral of silence a theory of public opinion. J Commun. (1974) 24:43–51. doi: 10.1111/J.1460-2466.1974.TB00367.X

[54] Sohn D. Spiral of silence in the social media era: a simulation approach to the interplay between social networks and mass media. Commun Res. (2022) 49:139–66. doi: 10.1177/0093650219856510

[55] Gearhart S Zhang W. Gay bullying and online opinion expression: testing spiral of silence in the social media environment. Soc Sci Comput Rev. (2014) 32:18–36. doi: 10.1177/0894439313504261

[56] Hakobyan A. Digitalization of communication and the spiral of silence theory. Wisdom. (2020) 14:19–30. doi: 10.24234/wisdom.v14i1.312

[57] Simpson C. Elisabeth Noelle-Neumann's “spiral of silence” and the historical context of communication theory. J Commun. (1996) 46:149–71. doi: 10.1111/J.1460-2466.1996.TB01494.X

[58] Lee W Detenber BH Willnat L Aday S Graf J. A cross-cultural test of the spiral of silence theory in Singapore and the United States. Asian J Commun. (2004) 14:205–26. doi: 10.1080/0129298042000256758

[59] Salmon CT Neuwirth K. Perception of opinion ‘climates’ and willingness to discuss the issue of abortion. Journal Q. (1990) 67:567–77. doi: 10.1177/107769909006700312

[60] Kushin MJ Yamamoto M Dalisay F. Societal majority, Facebook, and the spiral of silence in the 2016 US presidential election. Soc Med Soc. (2019) 5:2056305119855139. doi: 10.1177/2056305119855

[61] Tsfati Y. Media skepticism and climate of opinion perception. Int J Public Opin Res. (2003) 15:65–82. doi: 10.1093/IJPOR/15.1.65

[62] Matthes J Knoll J von Sikorski C. The “spiral of silence” revisited: a meta-analysis on the relationship between perceptions of opinion support and political opinion expression. Commun Res. (2018) 45:3–33. doi: 10.1177/0093650217745429

[63] Ross B Pilz L Cabrera B Brachten F Neubaum G Stieglitz S. Are social bots a real threat? An agent-based model of the spiral of silence to analyse the impact of manipulative actors in social networks. Eur J Inf Syst. (2019) 28:394–412. doi: 10.1080/0960085X.2018.1560920

[64] Short J Williams E Christie B. The Social Psychology of Telecommunications, 19(4), 451–484. London: Wiley (1976).

[65] Biocca F. Harms C. Gregg J. (2001). The Networked Minds Measure of Social Presence: Pilot Test of the Factor Structure and Concurrent Validity. Paper Presented at the 4th Annual International Workshop on Presence, Philadelphia, PA. Available at: https://www.researchgate.net/publication/200772411.

[66] Gao W Liu Z Li J. How does social presence influence SNS addiction? A belongingness theory perspective. Comput Hum Behav. (2017) 77:347–55. doi: 10.1016/J.CHB.2017.09.002

[67] Ridings CM Gefen D. Virtual community attraction: why people hang out online. Journal of computer-mediated. Communication. (2004) 10:JCMC10110. doi: 10.1111/J.1083-6101.2004.TB00229.X

[68] Shanthi A. Thayalan X. Heng L. T. Arumugam N. (2019). Social Presence in Virtual Communication to Foster on-Line Guanxi. Paper Presented at the 2019 IEEE 10th Control and System Graduate Research Colloquium (ICSGRC), Shah Alam, (pp.104–109).

[69] Lin X Sarker S Featherman M. Users’ psychological perceptions of information sharing in the context of social media: a comprehensive model. Int J Electron Commer. (2019) 23:453–91. doi: 10.1080/10864415.2019.1655210

[70] Vyncke B. Perko T. Van Gorp B. (2015). Influence of Mass Media Channels on Health-Related Risk Perception: The Case of Fukushima. Paper Presented at the RICOMET 2015, Brdo Castle. Available at: https://lirias.kuleuven.be/1860069?limo=0.

[71] Yim MS Vaganov PA. Effects of education on nuclear risk perception and attitude: theory. Prog Nucl Energy. (2003) 42:221–35. doi: 10.1016/S0149-1970(03)80010-0

[72] Ayran G Çevik Özdemir HN Yaman E. The effect of risk perception, mask use, and social distance behavior on perceived stress in the COVID-19 process: a sectional study. J Child Adolescent Psychiatric Nurs. (2023) 36:145–54. doi: 10.1111/jcap.12409 36705273

[73] Qian D Li O. The relationship between risk event involvement and risk perception during the COVID-19 outbreak in China. Appl Psychol Health Well Being. (2020) 12:983–99. doi: 10.1111/aphw.12219 32829535 PMC7461204

[74] Chung M Lim YS. When health organization answers the question: differential effects of dialogic messages in website and twitter through social presence and psychological distance. Health Commun. (2022) 37:685–95. doi: 10.1080/10410236.2020.1864098 33356904

[75] Biocca F Harms C Burgoon JK. Toward a more robust theory and measure of social presence: review and suggested criteria. Presence Teleop Virt. (2003) 12:456–80. doi: 10.1162/105474603322761270

[76] Zhang D Lowry PB Zhou L Fu X. The impact of individualism - collectivism, social presence, and group diversity on group decision making under majority influence. J Manag Inf Syst. (2007) 23:53–80. doi: 10.2753/MIS0742-1222230404

[77] China Internet Network Information Center. (2022). The 50th Statistical Report on China’s Internet Development. Available at: http://www.cnnic.net.cn/NMediaFile/2022/0926/MAIN1664183425619U2MS433V3V.pdf

[78] Lin CA Lagoe C. Effects of news media and interpersonal interactions on H1N1 risk perception and vaccination intent. Commun Res Rep. (2013) 30:127–36. doi: 10.1080/08824096.2012.762907

[79] Salwen MB Lin C Matera FR. Willingness to discuss ‘official English’: a test of three communities. Journal Q. (1994) 71:282–90. doi: 10.1177/107769909407100203

[80] Zhang X Liu S Deng Z Chen X. Knowledge sharing motivations in online health communities: a comparative study of health professionals and normal users. Comput Human Behav. (2017) 75:797–810. doi: 10.1016/j.chb.2017.06.028

[81] Shang L Zhou J Zuo M. Understanding older adults' intention to share health information on social media: the role of health belief and information processing. Internet Res. (2021) 31:100–22. doi: 10.1108/INTR-12-2019-0512

[82] Kline RB. Principles and Practice of Structural Equation Modeling. 4th ed. New York: Guilford Press (2016).

[83] Fornell C Larcker DF. Evaluating structural equation models with unobservable variables and measurement error. J Mark Res. (1981) 18:39–50. doi: 10.1177/002224378101800104

[84] Hair JF Black WC Babin BJ Anderson RE Tatham RL. Multivariate Data Analysis. 6th ed. New Jersey: Prentice Hall International (2006).

[85] Salisbury WD Chin WW Gopal A Newsted PR. Research report: better theory through measurement—developing a scale to capture consensus on appropriation. Inf Syst Res. (2002) 31:1383–405. doi: 10.1108/INTR-06-2019-0260

[86] Nitzl C Roldan JL Cepeda G. Mediation analysis in partial least squares path modelling, helping researchers discuss more sophisticated models. Ind Manag Data Syst. (2016) 116:1849–64. doi: 10.1108/IMDS-07-2015-0302

[87] Chung N Han H Koo C. Adoption of travel information in user-generated content on social media: the moderating effect of social presence. Behav Inform Technol. (2015) 34:902–19. doi: 10.1080/0144929X.2015.1039060

[88] Himelboim I Xiao X Lee DKL Wang MY Borah P. A social networks approach to understanding vaccine conversations on twitter: network clusters, sentiment, and certainty in HPV social networks. Health Commun. (2020) 35:607–15. doi: 10.1080/10410236.2019.1573446 31199698

[89] Park M. Information sharing to promote risky health behavior on social media. J Health Commun. (2019) 24:359–67. doi: 10.1080/10810730.2019.1604914 31033412

[90] Gan C Li H. Understanding the effects of gratifications on the continuance intention to use WeChat in China: a perspective on uses and gratifications. Comput Hum Behav. (2018) 78:306–15. doi: 10.1016/J.CHB.2017.10.003

[91] Hassanein K Head M Ju C. A cross-cultural comparison of the impact of social presence on website trust, usefulness and enjoyment. Int J Electron Bus. (2009) 7:625–41. doi: 10.1504/IJEB.2009.029050

[92] Kane GC Fichman RG Gallaugher J Glaser J. Community relations 2.0. Harv Bus Rev. (2009) 87:45–50. PMID: 19891388

[93] Treem JW Leonardi PM. Social media use in organizations: exploring the affordances of visibility, editability, persistence, and association. Ann Int Commun Assoc. (2013) 36:143–89. doi: 10.1080/23808985.2013.11679130

[94] Stone RN Grønhaug K. Perceived risk: further considerations for the marketing discipline. Eur J Mark. (1993) 27:39–50. doi: 10.1108/03090569310026637

[95] Chaudhry I Gruzd A. Expressing and challenging racist discourse on Facebook: how social media weaken the “spiral of silence” theory. Policy Internet. (2020) 12:88–108. doi: 10.1002/poi3.197

[96] Glynn CJ Hayes AF Shanahan J. Perceived support for one's opinions and willingness to speak out: a meta-analysis of survey studies on the "spiral of silence". Public Opin Q. (1997) 61:452–63. doi: 10.1086/297808

[97] Han Y. Research on the anti-silence spiral phenomenon in the transmission of internet public opinion—take the incident of “corporal punishment of a girl with asthma in Guangzhou to cause hematemesis” as an example. Open J Soc Sci. (2021) 9:359–66. doi: 10.4236/jss.2021.97026

[98] Balakrishnan V Ng KS Rahim HA. To share or not to share-the underlying motives of sharing fake news amidst the covid-19 pandemic in Malaysia. Technol Soc. (2021) 66:101676. doi: 10.1016/j.techsoc.2021.101676 36540782 PMC9754941

[99] Ball C Huang KT Francis J. Virtual reality adoption during the covid-19 pandemic: a uses and gratifications perspective. Telematics Inform. (2021) 65:101728. doi: 10.1016/j.tele.2021.101728 PMC8520656 34887619

[100] Benkler Y Roberts H Faris R Solow-Niederman A Etling B. Social mobilization and the networked public sphere: mapping the SOPA-PIPA debate. Polit Commun. (2015) 32:594–624. doi: 10.1080/10584609.2014.986349

[101] Entradas M. In science we trust: the effects of information sources on COVID-19 risk perceptions. Health Commun. (2022) 37:1715–23. doi: 10.1080/10410236.2021.1914915 33941007

[102] Huang Y Yang C. A metacognitive approach to reconsidering risk perceptions and uncertainty: understand information seeking during COVID-19. Sci Commun. (2020) 42:616–42. doi: 10.1177/1075547020959818

[103] Liu M Zhang H Huang H. Media exposure to COVID-19 information, risk perception, social and geographical proximity, and self-rated anxiety in China. BMC Public Health. (2020) 20:1–8. doi: 10.1186/s12889-020-09761-8 33148201 PMC7609828

[104] Masullo GM Lu S Fadnis D. Does online incivility cancel out the spiral of silence? A moderated mediation model of willingness to speak out. New Media Soc. (2021) 23:3391–414. doi: 10.1177/1461444820954194

