# Supplementary figures and images for: How does social presence influence public crisis information sharing intention? Situational pressure perspective
Source: Front Public Health. 2023 Jul 11;11:1124876. doi: 10.3389/fpubh.2023.1124876 (PMC10367105; doi:10.3389/fpubh.2023.1124876)

confirmatory factor analyses


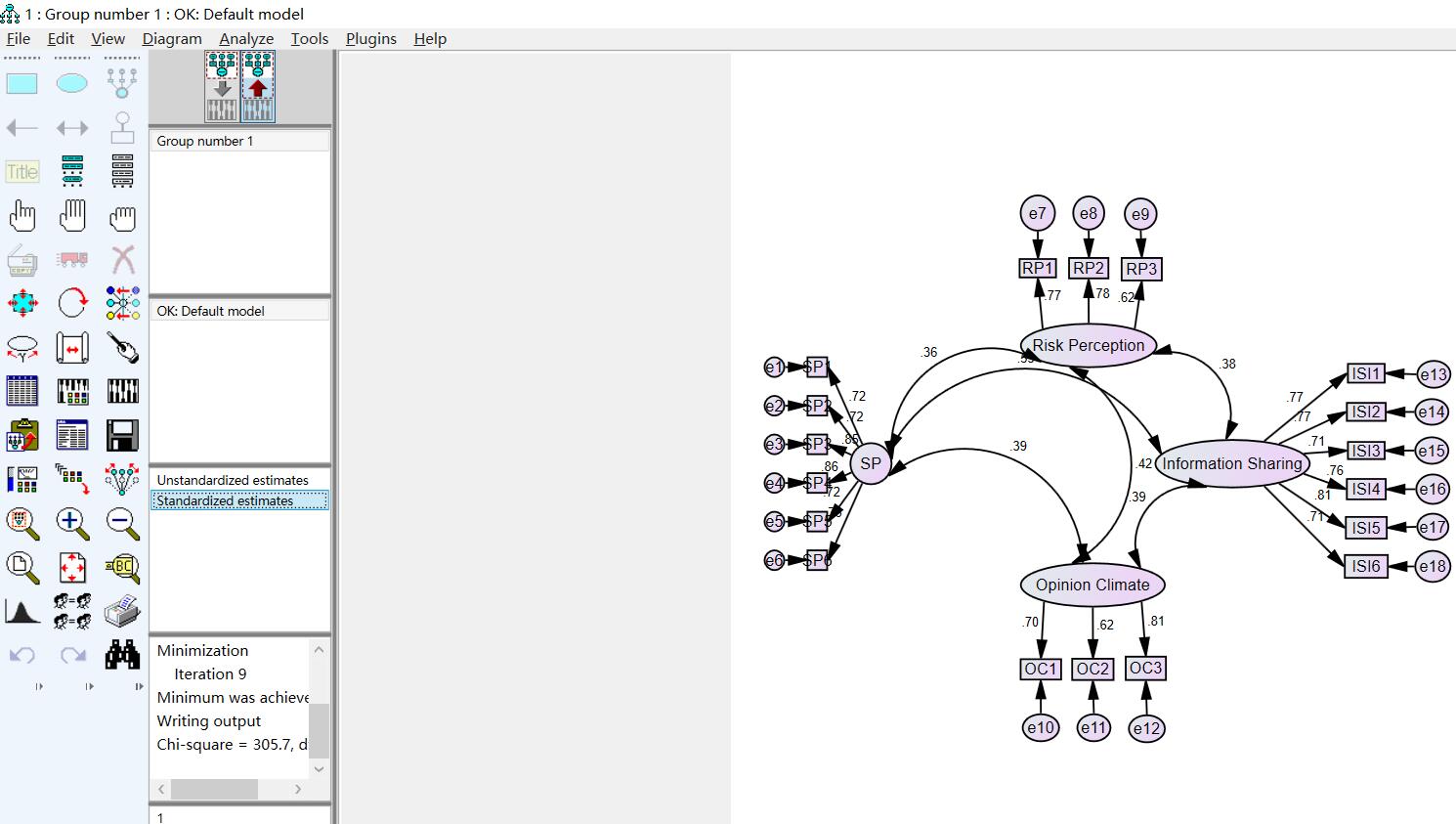


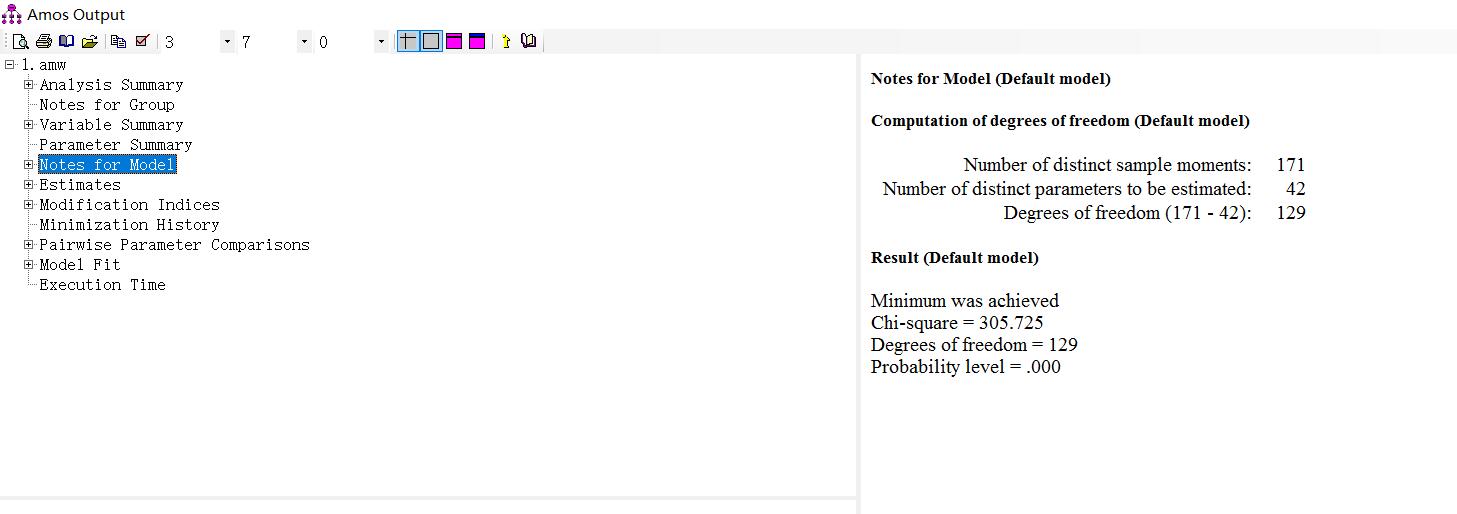


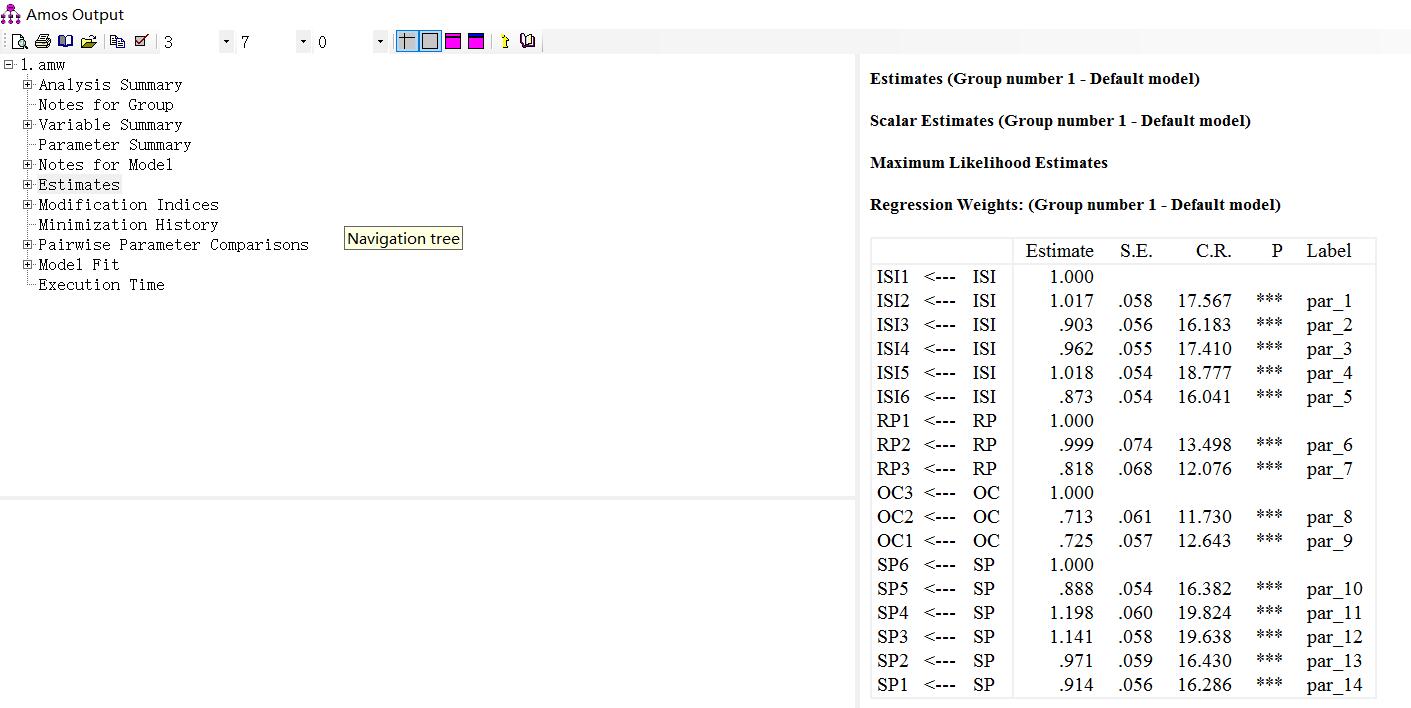


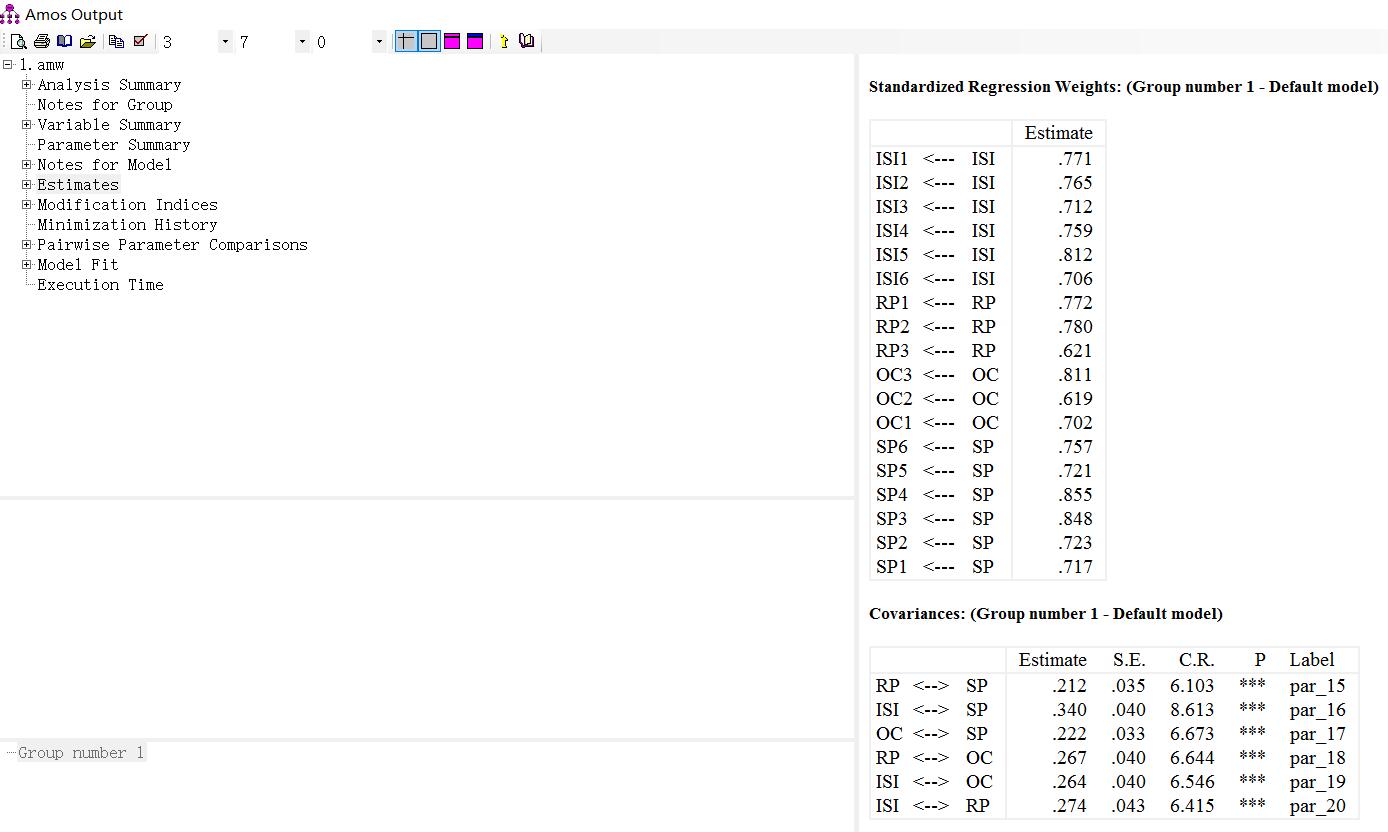


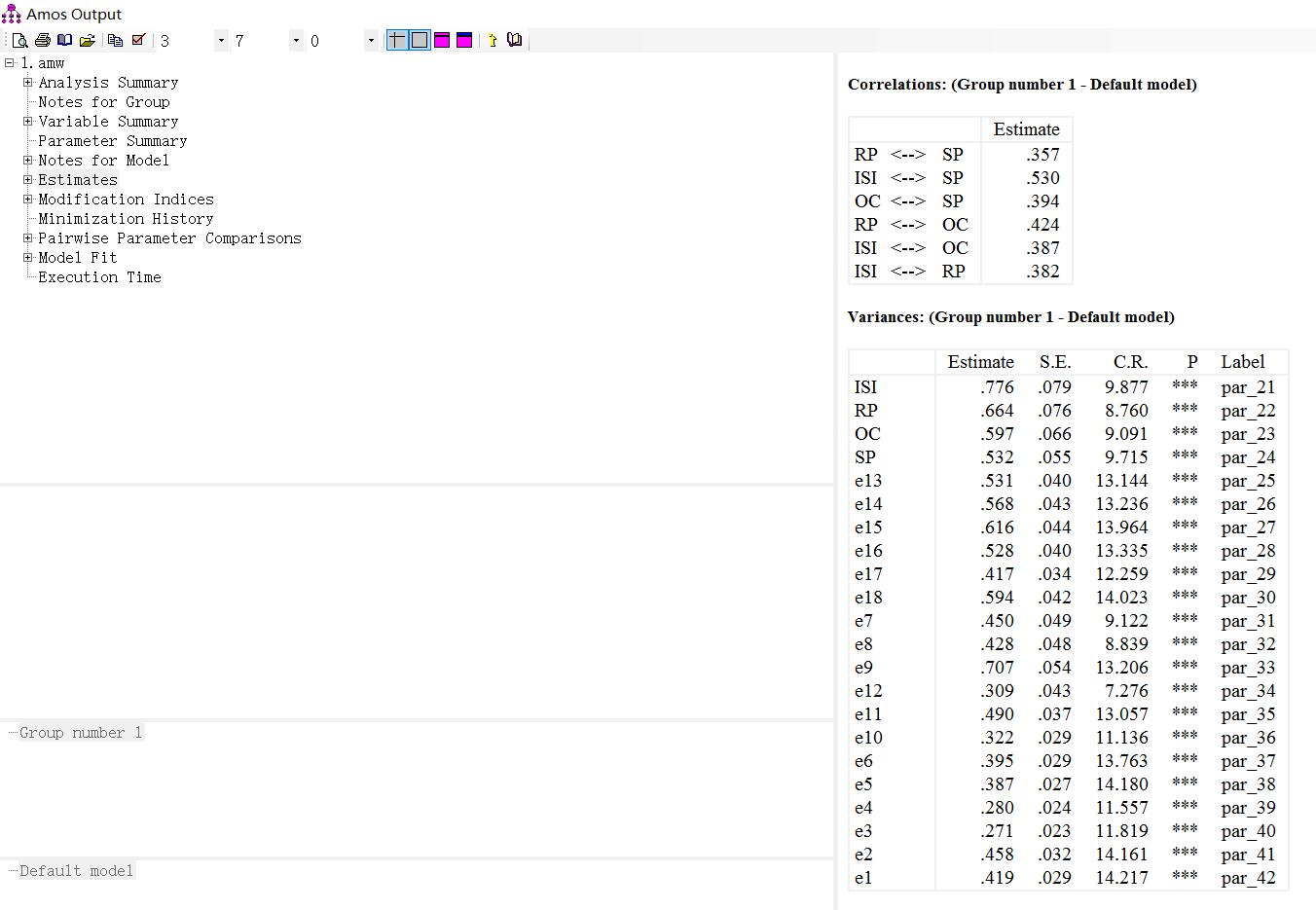


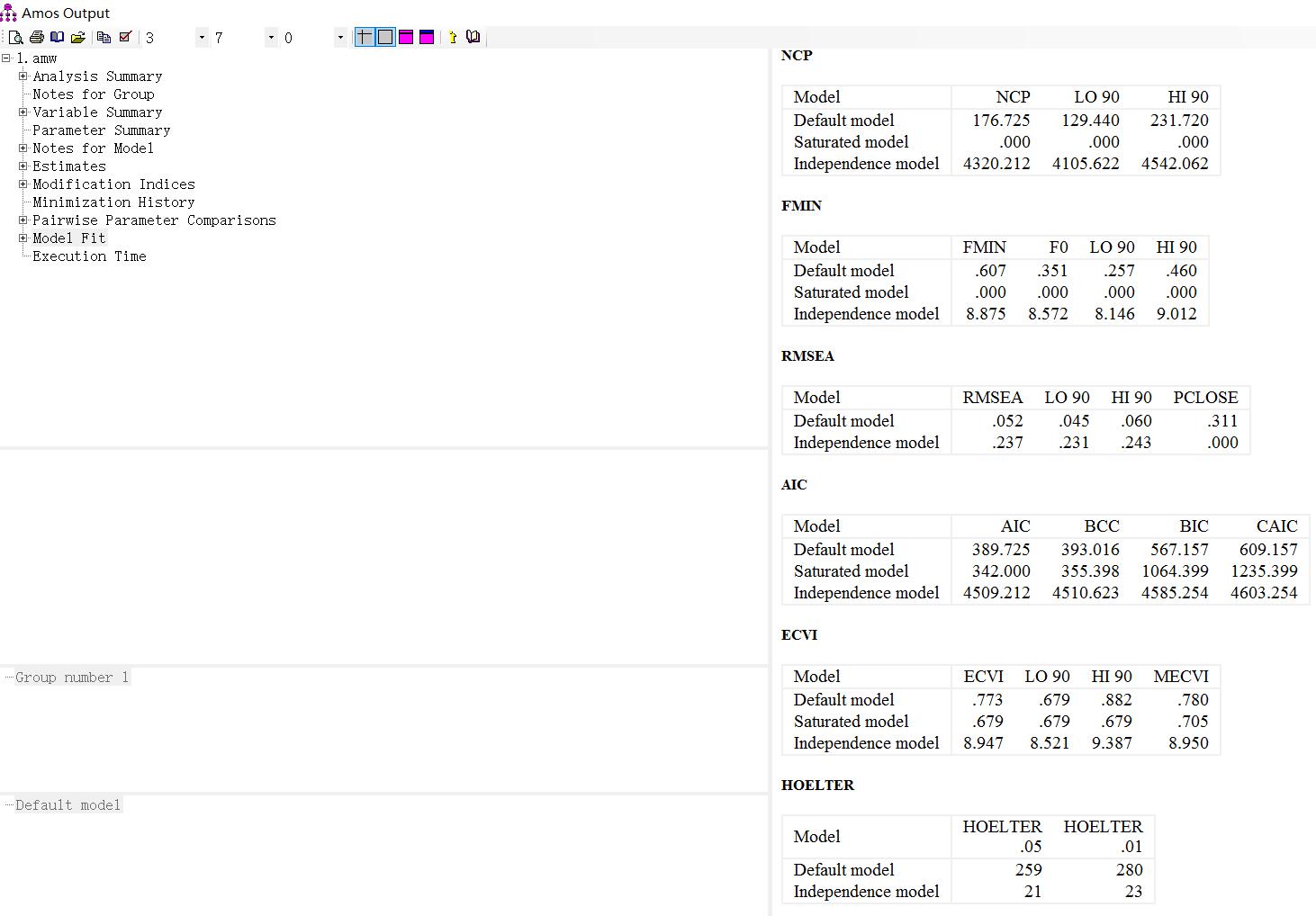

Supplement: Supplementary file 2 [file Data_Sheet_1.docx]
